# Differential Autoregulation of Serotonin Secretion at Different Structures of Serotonergic Neurons

**DOI:** 10.3390/ijms27031150

**Published:** 2026-01-23

**Authors:** Citlali Trueta, Montserrat G. Cercós

**Affiliations:** Departamento de Neurofisiología, Instituto Nacional de Psiquiatría Ramón de la Fuente Muñiz, Ciudad de México 14370, Mexico; montse@inprf.gob.mx

**Keywords:** serotonin, autoregulation, presynaptic terminal, somatic secretion, autoinhibition, feedback, autoreceptor, raphe nuclei, leech

## Abstract

Serotonin (5-HT) performs a wide range of neuromodulatory actions in the nervous system, including the regulation of the neurons that release it, by activation of several types of autoreceptors that modulate their electrical activity, as well as its own release. 5-HT neurons release serotonin in different manners from different subcellular structures, including the presynaptic terminals, the somatodendritic region and the axons. The different releasing structures of the same neurons have different types of autoreceptors, which exert differential auto-regulatory effects. Here we critically review the evidence of serotonergic autoregulation, both in mammals and in invertebrates, with particular emphasis on studies of serotonergic Retzius neurons of the leech, which have been a model for detailed studies of serotonin secretion from different neuronal structures. In these neurons serotonin produces different and even opposite effects on different releasing structures, such as the presynaptic terminals and the soma, through activation of different types of autoreceptors, thus increasing the specialization of the mechanisms that regulate exocytosis from each site. The differential autoregulation of serotonin release from different structures enables a single neuron to exert a variety of different functions in the nervous system.

## 1. Introduction

5-hydroxytryptamine (5-HT), named serotonin for its first known function, regulating the tone of blood vessels, is an important neurotransmitter that regulates mood, cognition and a variety of physiological processes, mostly by producing a wide variety of neuromodulatory functions in the nervous system, many of which are presented in this Special Issue. In addition to modulating the activity and functions of other neurons, one of the essential aspects of serotonergic function is autoregulation, the process by which serotonin modulates its own release, as well as the electrical activity of the neurons that release it, by activating autoreceptors on these same neurons.

A striking feature of serotonergic systems is that they are formed by relatively small numbers of neurons [1]. Yet, serotonin regulates a surprisingly wide variety of functions in the nervous system, from the generation of rhythmic motor patterns to very complex functions, such as regulation of attention or mood [2]. This may be explained by the fact that serotonergic(5-HT) neurons release this transmitter in different manners from different compartments of their structure, thus behaving as multifunctional cells. At synaptic terminals serotonin is released in small amounts and produces fast, short-lived and local effects that regulate fast processes in neural circuits; on the other hand, the soma of these neurons releases large amounts of serotonin in a slow and long-lasting manner [3,4,5], which may produce long-term paracrine modulation of large neuronal populations. Serotonin is also released extrasynaptically from the axonal shafts or varicosities [6], probably producing paracrine modulation of local circuits throughout the nervous system. Serotonin release at each of these neuronal compartments is finely and differentially regulated by different intracellular mechanisms, and autoregulation plays a key role in this compartmentalization.

Another feature worth noting is that the functions of serotonin have been highly conserved along the phylogenetic scale. Serotonin regulates rhythmic motor patterns, social and sexual behavior, along with many other functions, from invertebrates to mammals [2]. Serotonergic autoregulation also occurs throughout the animal kingdom and has been studied both in vertebrates and in invertebrates. However, while the complexity of the vertebrate nervous system has posed a challenge to understanding how serotonin regulates its own release, the simpler nervous systems of invertebrates, with large neurons at stereotyped locations and their regeneration capabilities, have allowed for directly studying the autoregulatory effects of serotonin on the electrical activity of 5-HT neurons and on its own release from different neuronal structures.

Here we review our current knowledge about serotonergic autoregulation by critically revising the evidence of different autoregulatory effects in serotonergic neurons of vertebrates and invertebrates, showing that different types of autoreceptors are localized at different secretory structures and exert different regulatory actions on neuronal activity and on serotonin release.

## 2. Serotonergic Autoregulation in Vertebrates

In the vertebrate central nervous system, the somata of 5-HT neurons are entirely located in a few nuclei in the brainstem, known as the raphe nuclei, of which the dorsal raphe nucleus (DRN) is the most studied. The axons of these neurons, however, innervate all areas of the brain and spinal cord [7]. Already in the decade of 1970, it became evident that 5-HT neurons are subject to feedback regulation, since it was shown that the activity of 5-HT neurons in the raphe nuclei decreased in response to manipulations that increase the availability of serotonin, or to application of exogenous serotonin or 5-HT agonists.

Initial work by Aghajanian and colleagues showed that inhibiting serotonin reuptake (by blocking the serotonin reuptake transporter), inhibiting serotonin degradation (by inhibiting monoamine oxidase) or increasing serotonin synthesis (by supplementation with its precursor, L-Tryptophan) decreased the firing rate of neurons in the raphe nuclei [8]. Application of serotonin or 5-HT agonists, either intravenously or directly to raphe nuclei, also decreased the firing rate of 5-HT neurons [9,10,11,12,13,14,15,16,17], as well as serotonin synthesis [18,19]. These manipulations also decrease serotonin release from nerve terminals in several brain areas [20,21]. All this evidence showed that serotonin exerted an inhibitory action on 5-HT neurons. Moreover, antidromic activation of 5-HT neurons resulted in a period of post-activation inhibition that depended on serotonin [22,23]. Furthermore, electrical stimulation within the DRN also produces a robust membrane hyperpolarization in 5-HT neurons that is inhibited by 5-HT antagonists [24], thus suggesting that this inhibition is produced by serotonin, released within the DRN.

These observations suggested that 5-HT neurons either express 5-HT autoreceptors on their surface or engage in circuits that establish feedback inhibitory connections onto the same neurons, thus participating in the regulation of their own electrical activity and, therefore, in the regulation of serotonin release. The complexity of the mammalian nervous system posed difficulties in discerning if the inhibitory effects of serotonin on 5-HT neurons are exerted directly onto these neurons through the activation of autoreceptors or through the action on interneurons that, in turn, connect back with 5-HT neurons. Extensive research in this field has shown that both indirect and direct mechanisms operate this autoregulatory activity of 5-HT neurons.

### 2.1. Indirect Feedback Regulation of 5-HT Neurons

5-HT neurons engage in several feedback loops by connecting to interneurons that, in turn, project back to them and regulate their activity and the release of serotonin. The 5-HT receptors participating in many of these feedback circuits are 5-HT_2A/C_ receptors, which are heteroreceptors located on other (non-5-HT) types of neurons.

**Figure 1:**
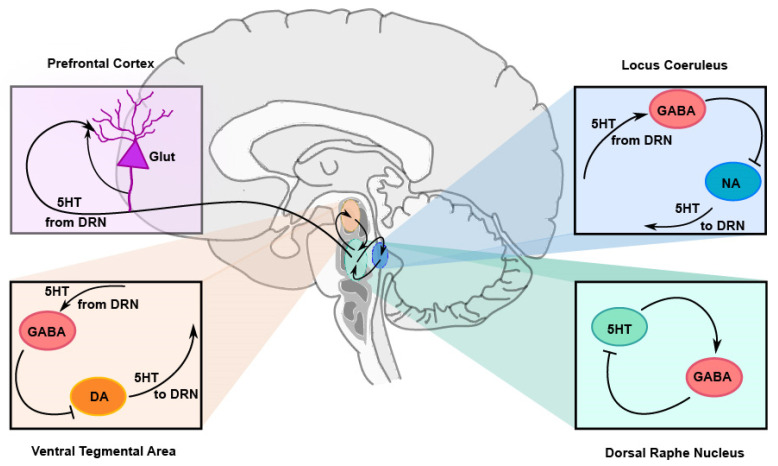
Indirect feedback loops of serotonergic autoregulation. 5-HT neurons, with their soma located in the raphe nuclei in the brainstem, project to multiple areas in the brain and spinal cord. Some of these areas project back to the raphe and exert a feedback regulation on 5-HT neurons. 5-HT projections from the dorsal raphe nucleus (DRN; green) to the ventral tegmental area (VTA; orange) activate GABAergic neurons, which, in turn, inhibit dopaminergic (DA) neurons in that area that project back to the DRN. Since DA activates 5-HT neurons, inhibition of DA neurons removes activation to 5-HT neurons, thus producing negative feedback. 5-HT projections to the locus coeruleus (LC; blue) activate GABAergic neurons, which, in turn, inhibit noradrenergic (NA) neurons in that area that project back to the DRN. Since NA activates 5-HT neurons, inhibition of NA neurons also produces negative feedback onto 5-HT neurons. In the prefrontal cortex (PFCx; purple), 5-HT projections activate glutamatergic (Glut) neurons, which synapse back onto 5-HT presynaptic terminals, increasing local serotonin release. 5-HT neurons in the DRN also activate GABAergic neurons within the DRN, which exert negative feedback onto 5-HT neurons. All these feedback loops are engaged by activation of 5-HT_2A/C_ heteroreceptors. Black lines represent axonal projections. Arrowheads (→) represent excitatory synapses; truncated lines (┤) represent inhibitory synapses.

Activation of 5-HT_2A/C_ receptors on GABAergic or glutamatergic neurons, either in the DRN or in other structures that project back to the DRN, indirectly regulates the release of serotonin (Figure 1). For example, activation of 5-HT_2A_ receptors on GABAergic neurons in the ventral tegmental area (VTA) increases the activity of GABAergic interneurons that establish inhibitory synapses with dopaminergic (DA) neurons, which, in turn, project back to the DRN. Since DA in the DRN enhances the activity of 5-TH neurons, the inhibition of DA neurons in the VTA contributes to decreased 5-TH activity. Something similar occurs with noradrenergic neurons in the locus coeruleus. On the other hand, glutamatergic pyramidal neurons of the medial prefrontal cortex receive serotonergic 5-HT_2A_ innervation and, in turn, synapse back onto the presynaptic 5-HT terminals, enhancing local serotonin release (For review see [25]).

In addition to this indirect feedback regulation, several types of 5-HT autoreceptors have been found on vertebrate 5-HT neurons, which exert direct autoregulatory effects, thus controlling the firing rate of these neurons and the release of serotonin. In the following sections, we review these effects, as well as the participation of different 5-HT autoreceptors.

### 2.2. Direct Autoregulation of 5-HT Neurons Through Autoreceptors

Several receptors, namely 5-HT_1A_, 5-HT_1D_, its rodent analogue 5-HT_1B_, and 5-HT_2B_, have been identified as autoreceptors, expressed by 5-HT neurons, that regulate their electrical activity and the release of serotonin. However, apparently different types of autoreceptors are specifically localized to different neuronal compartments. For example, it is generally accepted that the expression of the 5-HT_1A_ autoreceptor is restricted to the somatodendritic compartment of 5-HT neurons, whereas 5-HT_1B_ autoreceptors are located only on axons and presynaptic terminals [26,27,28,29].

In spite of the long-standing evidence that serotonin exerted feedback regulation onto 5-HT neurons, unequivocal evidence for the existence of autoreceptors was not found until 1990, when 5-HT_1A_ receptor immunoreactivity was found in 5-HT-immunopositive neurons by double immunolabeling [30]. 5-HT_1A_ autoreceptors in the raphe nuclei have been found exclusively on neuronal cell bodies and dendrites, mostly along extrasynaptic portions of their plasma membrane, i.e., not on dendritic postsynaptic terminals [28,29]. On the other hand, the 5-HT_1B_ receptor has been found in pre-terminal axons as well as in presynaptic terminals and is thought to regulate serotonin release presynaptically. Although the evidence for the presence of 5-HT_1B_ receptors specifically on serotonergic axons or terminals is scarce, Sari [27] labelled 5-HT neurons in the raphe with ^3^H-5-HT and showed that 5-HT_1B_ labelling by immunogold co-localizes with the autoradiographic localization of ^3^H-5-HT in axons in the substantia nigra, at the ultrastructural level. This observation complemented previous findings showing a decrease in binding sites for 5-HT_1B_ receptors in the substantia nigra after a lesion of the serotonergic system [31]. Together, these findings demonstrated that 5-HT_1B_ receptors are localized at the 5-HT nerve terminals of the raphe-nigral pathway, thus acting as autoreceptors. In addition, the expression of both 5-HT_1D_ and 5-HT_1B_ mRNA has been shown in neurons of raphe nuclei in rodents [32], as well as in post mortem studies in human brains [33], showing that 5-HT neurons express these autoreceptors. 5-HT_2B_ receptors have also been identified as autoreceptors, since their expression in 5-HT neurons in the DRN has been shown by single-cell RT-PCR [34]. This shows that at least part of the actions of serotonin are exerted directly through autoreceptors onto 5-HT neurons.

#### 2.2.1. 5-HT_1A_ Autoreceptors

The type of receptors producing autoregulatory effects on 5-HT neurons was delayed by the absence of selective antagonists, which, in the case of 5-HT_1A_ receptors, were not available until the middle of the decade of the 1990s. The development of WAY-100635 allowed the study of the specific effects of 5-HT_1A_ receptors on the function of 5-HT neurons. WAY-100635 prevents the decrease in the firing rate of 5-HT neurons produced by serotonin and by several agonists [35,36,37], supporting the inhibitory role of serotonin through 5-HT_1A_ autoreceptors. Although in some in vitro studies, WAY-100635 by itself has little or no effect on the firing rate of 5-HT neurons in the DRN [36,38], other studies of DRN slices have shown that most putative 5-HT neurons increase their firing rate upon superfusion with this selective 5-HT_1A_ antagonist [35]. Furthermore this antagonist was shown to increase the firing rate of 5-HT neurons in behaving cats during wakefulness, when 5-HT neurons typically display a relatively high level of activity, although not during sleep, when 5-HT neurons display little or no spontaneous activity [39]. Although this dependence on the arousal state has been called to question the function of 5-HT_1A_ autoreceptors as homeostatic controllers of the firing frequency of 5-HT neurons [40], the ineffectiveness of WAY-100635 to increase firing during sleep might suggest that other (extrinsic) inhibitory mechanisms maintain 5-HT neurons silent during sleep, while 5-HT_1A_ autoreceptors control the firing frequency of 5-HT neurons in the absence of these other inhibitory inputs.

**Figure 2:**
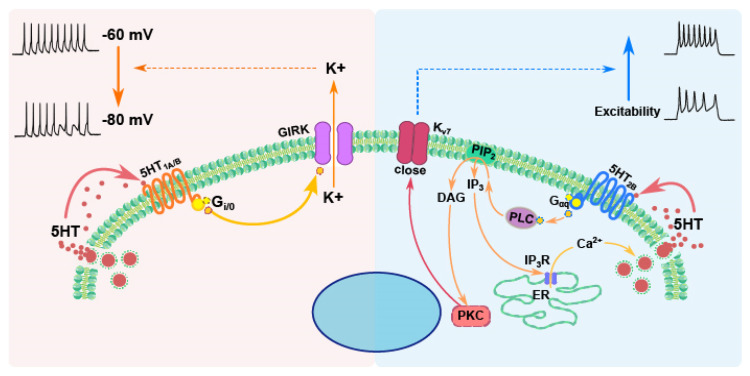
Intracellular pathways activated by 5-HT autoreceptors and their effects in 5-HT neurons of mammals. Left, orange shade: 5-HT_1A_ autoreceptors (orange) are coupled to Gi/o protein (yellow), which directly activates a G-protein-coupled inwardly rectifying potassium (GIRK) channel (purple). Opening of these channels hyperpolarizes the membrane and decreases the excitability of 5-HT neurons. 5-HT_1B_ autoreceptors, located primarily on axons, activate the same mechanism, possibly modulating the conduction of action potentials to the presynaptic terminals. Right, blue shade: 5-HT_2B_ autoreceptors (blue) couple to phospholipase C (PLC) through Gαq proteins. PLC breaks down Phosphatidylinositol bisphosphate (PIP_2_) into diacylglycerol (DAG) and inositol trisphosphate (IP_3_). DAG activates protein kinase C (PKC), which increases excitability through closing Kv7 potassium channels (red); IP_3_ activates receptors on the endoplasmic reticulum (ER) membrane, allowing the release of calcium (Ca^2+^) from this store to the cytoplasm, which increases serotonin exocytosis. Serotonin (small red dots) released from vesicles (large red dots) in the somatodendritic region of 5-HT neurons activates autoreceptors located in this region.

Short electrical stimulation in the DRN produces a transient outward current in 5-HT neurons (identified in mice expressing a fluorescent marker in these neurons) that leads to a transient decrease in firing that is blocked by the selective 5-HT_1A_ antagonist WAY-1000635 [41]. This shows that electrical stimulation of the raphe nuclei leads to serotonin release, which, in turn, has an autoinhibitory effect on 5-HT neurons through 5-HT_1A_ receptors. The inhibitory effects of 5-HT_1A_ receptors are mediated by the opening of G-protein-coupled inwardly rectifying potassium (GIRK) channels of the Kir3 family [17,24,42,43], which hyperpolarize the membrane and decrease neuronal excitability (Figure 2).

All this provides robust evidence that autoinhibition in the somatodendritic compartment of 5-HT neurons within the raphe nuclei is mainly mediated by 5-HT_1A_ autoreceptors, which, by activating outward potassium currents, decrease the firing rate of 5-HT neurons.

The activation of 5-HT_1A_ receptors by systemic agonists, such as 8-OH-DPAT or gepirone, also causes a reduction in serotonin release in 5-HT projection areas, such as the ventral hippocampus [44], showing that a decrease in the firing rate of 5-HT neurons leads to a decrease in serotonin release at axons and terminals.

5-HT_1A_ receptors also act as heteroreceptors in postsynaptic neurons in several areas of projection of 5-HT neurons, such as the hippocampus [28], where they mediate serotonin-induced hyperpolarization [45,46]. However, it is interesting that the mechanism of action of the selective antagonist WAY-100635 is different in auto- and heteroreceptors: it is a competitive antagonist in the DRN, while it acts as a partly noncompetitive antagonist in the hippocampus [35].

Since antagonists can block not only autoreceptors but also heteroreceptors, the use of antagonists can pose questions about the role of a specific receptor type as an autoreceptor. For this reason, the function of 5-HT_1A_ autoreceptors in the regulation of the firing frequency of 5-HT neurons has also been studied by developing a mouse strain with lower expression of 5-HT_1A_ receptors (1A-Low mice) specifically in 5-HT neurons (autoreceptors). 1A-Low mice have a higher firing rate in raphe neurons, supporting the autoinhibitory role of these autoreceptors on the firing frequency of 5-HT neurons [47].

The decrease in the activity of 5-HT neurons upon increases in 5-HT availability is likely the cause of the delayed onset of the antidepressant action of selective serotonin reuptake inhibitors (SSRI) or monoamine oxidase inhibitors [48]. With prolonged treatment the firing of 5-HT neurons gradually recovers, as a result of 5-HT_1A_ autoreceptor desensitization, which occurs through decreased coupling with potassium channels by Gi/o proteins, thus allowing SSRI to increase serotonin availability.

#### 2.2.2. 5-HT_1B_ Autoreceptors

The presence of 5-HT_1B_ mRNA in 5-HT neurons of raphe nuclei [32,49] demonstrates that 5-HT neurons express these receptors as autoreceptors. However, extensive autoradiographic and immunoautoradiographic studies showed that little or no presence of 5-HT_1B_ binding sites (receptors) can be detected in the raphe nuclei of rodents [26,28,32], suggesting that the protein is not localized in the somatodendritic region of 5-HT neurons, and that these autoreceptors are rather related to presynaptic regulation of serotonin release. 5-HT_1B_ receptors have been found in different regions of the CNS where raphe neurons project, including the substantia nigra, globus pallidus, dorsal subiculum, hippocampus and the prefrontal cortex, where they have been shown to decrease serotonin release (see below). These receptors, however, are mostly localized on pre-terminal regions of unmyelinated axons and not directly on the synaptic terminals [26,28], thus suggesting that they may not modulate neurotransmitter release directly at the presynaptic terminals, but rather alter axonal conduction of action potentials before they arrive at the terminals [28]. The presence of these receptors in axonal regions external to the synapses in different neurons is also an indication that serotonin acts through diffuse extrasynaptic communication in the nervous system [28,50].

Like 5-HT_1A_, 5-HT_1B_ receptors are, in general, inhibitory. Different intracellular pathways have been identified to be regulated by these receptors in different types of cells. They are negatively coupled to adenylyl cyclase through Gαi, and their activation leads to an inhibition of cAMP accumulation [51]. They can also be coupled to the activation of the MAP-kinase, as well as to the pERK and p70 signaling systems [52]. 5-HT_1B_ receptors increase the activity calcium-dependent potassium channels through IP_3_-mediated intracellular calcium (Ca^2+^) release [53]. In neurons, these receptors, like 5-HT_1A_ receptors, activate potassium (GIRK) channels (Figure 2) [54,55], thus producing a hyperpolarization and a decrease in electrical activity or neurotransmitter release. At the Calyx of Held of immature rats, 5-HT_1B_ receptors inhibit transmitter release by inhibiting voltage-gated Ca^2+^ channels [56].

The inhibitory effect of 5-HT_1B_ receptors on serotonin release in 5-HT projection areas has been widely documented. For example, serotonin release in the hippocampus is inhibited by superfusion with serotonin, and this inhibition is mimicked by 5-HT_1B_ agonists and competitively blocked by 5-HT_1B_ antagonists, showing that it is mediated by 5-HT_1B_ receptors [57]. Microdialysis of 5-HT_1B_ agonists such as RU-24969 or CP-94253 in the anterior hypothalamus also decreased the release of serotonin [58], while infusion of methiotepin, a 5-HT antagonist, increased release and prevented RU-24969-induced decrease, suggesting that terminal 5-TH_1B_ autoreceptors decrease serotonin release [59]. Serotonin efflux in the frontal cortex is also decreased by 5-HT_1B_ agonists and increased by 5-HT_1B_ antagonists [60], supporting the inhibitory role of these autoreceptors on axonal serotonin release in this area. The administration of selective 5-HT_1B_ receptor agonists in rats decreased 5-HT release in several 5-HT projection areas (substantia nigra pars reticulata, ventral pallidum, lateral habenula, suprachiasmatic nucleus, hippocampus and globus pallidus), and this effect was prevented by the 5-HT_1B_ antagonist SB-224289 [61]. Systemic administration of agonists of 5-HT_1B_ receptors that pass the hematoencephalic barrier also produces a dose-dependent decrease in hippocampal and striatal 5-HT output [20,44,62]. In the same line, serotonin release stimulated by high potassium from globus pallidus synaptosomes in vitro was attenuated by a 5-HT_1B_ agonist [63].

5-HT_1B_ receptors also increase the reuptake of serotonin via the serotonin transporter, as shown by studies using high-speed chronoamperometry to measure clearance of 5-HT from the CA3 region of the hippocampus in vivo, where the 5-HT_1B_ receptor antagonist cyanopindolol prolonged the clearance of serotonin from the extracellular fluid [64,65]. This occurs in wild-type mice, but not in 5-HT_1B_−/− mice, and is diminished in 5-HT_1B_+/− mice, indicating that the 5-HT_1B_ receptor is necessary for this inhibition of serotonin clearance [66]. It has also been shown that 5-HT neurons of 5-HT_1B_−/− mice are less sensitive than wild-type mice to citalopram, a selective serotonin reuptake inhibitor, which is less effective at inhibiting the firing rate of DRN neurons in 5-HT_1B_−/− mice than in wild-type mice, and this difference is reduced by administrating a 5-HT_1B_ antagonist to wild-type mice, an effect that can be accounted for by a higher expression of the serotonin transporter in knockout mice [67]. All this is consistent with SERT being under positive regulatory control of 5-HT_1B_ autoreceptors. Thus, 5-HT_1B_ autoreceptors decrease serotonin availability in the extracellular fluid both by decreasing serotonin release and by increasing its reuptake.

5-HT_1B_ receptors are also present as heteroreceptors in presynaptic terminals of non-5-HT neurons, where they reduce the release of other neurotransmitters, including noradrenaline [68,69], GABA [70], dopamine [71] or acetylcholine (ACh) [72,73]. In the nucleus accumbens serotonin causes a long-term depression of glutamatergic transmission through 5-HT_1B_ receptors located at glutamatergic presynaptic terminals [74]. In the raphe nuclei 5-HT and GABAergic neurons are reciprocally innervated, and there is a reciprocal influence between these two neuron types [75].

It is interesting that the potency of selective 5-HT_1B_ agonists, such as CP93-129, or antagonists, such as SB224289, is different in auto and heteroreceptors, acting at much lower concentrations on autoreceptors to modulate presynaptic serotonin release than on heteroreceptors, modulating, for example, dopamine release in the striatum or acetylcholine release in the hippocampus [71].

#### 2.2.3. 5-HT_2B_ Autoreceptors

5-HT_2B_ receptors can also function as autoreceptors located on 5-HT neurons in the raphe nuclei of the brain. Unlike 5-HT_1A_ and 5-HT_1B_ autoreceptors, which, as shown above, generally provide negative feedback, 5-HT_2B_ autoreceptors appear to positively modulate serotonergic activity.

5-HT_2B_ receptors signal primarily through Gαq protein to activate phospholipase C (PLC), which hydrolyzes Phosphatidyl-inositol bisphosphate (PIP_2_) into two second messengers: inositol trisphosphate (IP_3_) and diacylglycerol (DAG) [76]. IP_3_ binds to receptors on the endoplasmic reticulum, triggering the release of intracellular Ca^2+^, while DAG activates protein kinase C (PKC), increasing neuronal excitability by closing Kv7 potassium channels [77] (Figure 2), thus increasing membrane resistance. In this way, 5-HT_2B_ autoreceptors can directly influence calcium signaling and enhance serotonergic neuronal activity.

The 5-HT_2B_ receptor agonist BW-723C86 increased the firing rate of 5-HT neurons in mouse brain slices and counteracted the 5-HT_1A_-dependent reduction in the firing rate in vivo. In addition, viral overexpression of the 5-HT_2B_ receptor specifically in 5-HT neurons increased their excitability, while 5-HT neurons of mice with a conditional genetic ablation of 5-HT_2B_ receptors specifically from these neurons had a lower firing rate than control neurons [78], showing that these receptors increase the firing rate of 5-HT neurons. Consistently, local infusion of BW-723C86 into the DRN by microdialysis increased extracellular serotonin, an effect that was blocked by the highly selective 5-HT_2B_ receptor antagonist RS127445 [79]. 5-HT neurons in 5-HT_2B_ conditional knockout animals also show exaggerated 5-HT_1A_ receptor-mediated responses, reflected by greater hyperpolarization, suggesting that 5-HT_2B_ receptors may play a role in counteracting the inhibitory influence of 5-HT_1A_ autoreceptors to help maintain the proper balance between inhibition and excitation within the serotonergic network.

In 5-HT-containing neurons of the guinea pig dorsal raphe nucleus in vitro, bath-applied serotonin evoked hyperpolarization and inhibition of electrical activity, which appeared to involve the activation of an inwardly rectifying K^+^ conductance and was mediated by 5-HT_1A_ autoreceptors. However, in the presence of the selective 5-HT_1A_ antagonist WAY-100635, serotonin induced a depolarizing, excitatory response associated with an increase in the input resistance of the neuron, likely due to the suppression of a K^+^ conductance. This effect was significantly reduced or abolished by 5-HT_2_ antagonists, indicating it is mediated by these receptors [80]. Interestingly, 5-HT_2B_ receptors have been shown to form complexes with 5-HT_1A_ receptors in 5-HT neurons in the raphe to regulate their excitability. While co-expression of both receptors leads to a decrease in the firing rate through activation of GIRK channels upon activation of 5-HT_1A_ receptors, the absence of 5-HT_2B_ receptors leads to an increase in the firing rate when 5-HT_1A_ receptors are activated, through inhibition of a Ca^2+^-activated potassium channel [81]. Thus, the clustering of these two autoreceptors seems to be crucial for the autoregulation of 5-HT neurons, finely tuning their excitability.

In addition, 5-HT_2B_ autoreceptors interact with the serotonin transporter to regulate the uptake of serotonin. In cultured 5-HT-like cells and primary raphe 5-HT neuron cultures, 5-HT_2B_ receptor activation produces the phosphorylation of the serotonin transporter and decreases its function [82], thus increasing the extracellular concentration of serotonin, an effect that would oppose the effects of 5-HT_1B/1D_ autoreceptors on serotonin reuptake. In fact, stimulation with agonists of 5-HT_2B_ receptors induces both behavioral and neurogenic responses similar to those produced by SSRI, while the SSRI-induced increase in extracellular serotonin concentrations in the hippocampus, as well as the long-term behavioral and neurogenic responses, are abolished by either genetic or pharmacological inactivation of 5-HT_2B_ receptors, thus showing that 5-HT_2B_ receptors are required for the antidepressant effects of SSRI [83]. Other in vivo studies have further confirmed that 5-HT_2B_ receptors contribute to the behavioral and physiological effects of drugs that target the serotonin transporter and increase the release of serotonin, such as MDMA (ecstasy) and dexfenfluramine [79,84].

### 2.3. How Do 5-HT Neurons Regulate Themselves?

The studies showing serotonergic autoinhibition in the raphe nuclei were initially interpreted by envisioning that 5-HT neurons had axonal recurrent collaterals that returned to the raphe nuclei and innervated 5-HT neurons. However, such recurrent collaterals have not been demonstrated [48,85]. Subsequent work using electron microscopy showed the presence of secretory vesicles and of serotonin in the dendrites of 5-HT cells [6,86], thus suggesting that serotonin could be released from these neuronal compartments. Furthermore, studies using cyclic voltammetry have shown stimulus-evoked release of serotonin in the DRN [87,88,89,90], further confirming extrasynaptic serotonin release from the somatodendritic region of 5-HT neurons. All this led to the suggestion that autoinhibition could be mediated via extrasynaptic release of serotonin. Extrasynaptic exocytosis from the soma of 5-HT neurons was initially shown in invertebrate neurons [5], and the release of serotonin from the soma as well as from dendrites in the dorsal raphe nucleus of mammals was later demonstrated by use of capacitance measurements and amperometry [91], as well as by multi-photon microscopy [3,92,93]. Although serotonin release from the soma can be activated by action potentials [93], neither electrical activity nor calcium transients in the soma backpropagate to the dendrites [91]. Nevertheless, both somatic and dendritic release can be stimulated by glutamate in the absence of action potentials. Dendritic release can be triggered both by AMPA and by NMDA and depends on the activation of L-type calcium channels [93]. Despite the high-voltage dependence of these channels, which makes their activation by sub-threshold graded potentials unlikely, the abundance in the dorsal raphe [94] of the Cav1.3 L-type channel isoform, which activates at negative potentials and can produce sustained currents [95], probably explains this finding.

All this evidence suggests that serotonin within the raphe nuclei is released by the soma and dendrites of 5-HT neurons in response to glutamatergic inputs, independently of the production of action potentials. Although the inhibitory responses elicited in the DRN neurons by electrical stimulation within the nucleus were initially described as “inhibitory postsynaptic potentials” and claimed to be “synaptic”, they have a long latency (47 ms), a strikingly slow time course of 1–2 s [24] and a strong dependence on diffusion [41]. Analyzing this evidence critically, in light of the demonstration that extrasynaptic release of serotonin occurs from the soma and dendrites of these neurons, and that extrasynaptic communication is a slow process, it is more likely that autoinhibition within the DRN is rather caused by somato-dendritic extrasynaptic release and not by release from synapses formed by the initially proposed “recurrent collaterals”. This idea is further supported by the observations that 5-HT_1A_ autoreceptors have been found on extrasynaptic areas of the cell bodies and dendrites of 5-HT neurons in the DRN [28,29,30].

It has been proposed that 5-HT_1A_ receptors regulate somatodendritic serotonin release, possibly not by directly regulating the firing frequency of 5-HT neurons, but by inhibiting the glutamate-induced secretion of serotonin from the soma or dendrites, which, as explained above, can occur independently of action potential firing and depends on NMDA receptors and on L-type calcium channels [40]. However, extensive evidence also shows that activation of 5-HT_1A_ and 5-HT_2B_ autoreceptors also regulates the firing rate of 5-HT neurons in the raphe nuclei (see above). While activation of 5-HT_1A_ autoreceptors decreases the firing rate, activation of 5-HT_2B_ autoreceptors increases it through the activation of PKC. Thus, the balance between the activation of these two types of autoreceptors finely tunes the firing rate of 5-HT neurons. It is noteworthy that the intracellular pathway activated by 5-HT_1A_ receptors is faster than that activated by 5-HT_2B_ receptors since, in the case of 5-HT_1A_ receptors, the Gi/o protein directly activates GIRK channels [17], while the pathway activated by 5-HT_2B_ receptors involves the activation of a longer intracellular cascade [96] (Figure 2). For this reason, it is more likely to observe an inhibition of 5-HT neurons when they are bathed by serotonin.

Despite the reported absence or little presence of 5-HT_1B_ receptors in the somatodendritic area of 5-HT neurons in the raphe nuclei [28,32], it has been shown that these receptors also exert an influence both on the firing rate of 5-HT neurons and on serotonin release within the raphe. However, the control of the firing rate of 5-HT neurons and of serotonin release in the raphe nuclei by 5-HT_1B_ receptors is controversial, since both excitatory and inhibitory effects have been observed. While an excitatory effect of 5-HT_1B_ agonists has been found on the firing rate of 5-HT neurons [61,67], the release of serotonin in the raphe nuclei (measured either by microdialysis or by cyclic voltammetry) is decreased by 5-HT_1B_ agonists, and this decrease is prevented by 5-HT_1B_ antagonists [61,97]. Since 5-HT_1B_ receptors are inhibitory [51,54,55,56], the increase in the firing rate observed in 5-HT neurons is likely mediated by 5-HT_1B_ heteroreceptors on GABAergic interneurons within the raphe nuclei, which, as explained above, have a reciprocal innervation with 5-HT neurons [75]. Serotonergic inhibition through 5-HT_1B_ receptors on these inhibitory interneurons would cause disinhibition of 5-HT neurons, which may explain the increase in the firing rate upon activation of 5-HT_1B_ receptors. On the other hand, the inhibitory effect of 5-HT_1B_ receptors on serotonin release in the raphe nuclei is consistent with the effect of these receptors on serotonin release from axons or presynaptic terminals in other brain areas and seems to be exerted directly on such release sites within the raphe nuclei. Taken together, these findings suggest that, in spite of the excitatory somatodendritic effects on the firing rate of 5-HT neurons, the activation of terminal 5-HT_1B_ autoreceptors can override these excitatory effects, ultimately reducing axonal serotonin release [98].

## 3. Serotonergic Autoregulation in Invertebrates: Opposite Effects on Different Secretory Structures in Retzius Neurons of the Leech

As can be deduced from the preceding sections, the study of serotonergic autoregulatory effects in mammals has been difficult and filled with controversies due to the complexity of the system. Invertebrates have simpler nervous systems, with smaller numbers of neurons of a larger size, which have been very useful to study basic neuronal mechanisms that are conserved throughout evolution. Work on such nervous systems has allowed for studying, in great detail, many aspects of neurophysiology, from the mechanisms underlying the production and conduction of the action potential [99] to the neuromodulation of neural circuits [100]. As with many subjects in neurobiology, the simpler nervous system of invertebrates has offered an alternative to study, in a more straightforward manner, the autoregulatory effect of serotonin on 5-HT neurons. Our work using Retzius neurons of the leech has shown how serotonin exerts different autoregulatory actions through different autoreceptors, differentially located at distinct secretory structures of the same neurons.

Invertebrate neurons have the experimental advantages of being large and easily identifiable, which makes them easily amenable to electrophysiological and imaging recordings. They can also be individually isolated and kept in culture, where they retain their physiological identity and continue to synthesize and release neurotransmitters [101]. Because of these advantages, the fine mechanisms regulating serotonin release have been studied in detail in leech serotonergic Retzius neurons, which release serotonin from presynaptic terminals [101], from the soma [5] and from the primary axon. The mechanisms responsible for serotonin release from these structures are intimately related to the particular autoregulation effects exerted by serotonin at each of them.

### 3.1. Synaptic Serotonin Release at Retzius Neurons

When plated in culture, making contact with an appropriate target neuron, such as a pressure sensory (P) cell, Retzius neurons form chemical synapses that have many advantages for studying synaptic serotonin release [102]. One of these advantages is that the neurons in culture are practically isopotential, and thus the electrical events in the presynaptic (as well as postsynaptic) terminals can be accurately recorded with an intracellular electrode in the soma of the neurons, without attenuation. Serotonin release from presynaptic terminals of cultured Retzius neurons displays the characteristics of release from classically studied synapses, such as the squid giant synapse or the neuromuscular junction [103]; it occurs in a quantal and calcium-dependent manner from small, clear vesicles [104]. As with transmitter release at any synapse, serotonin release from presynaptic terminals can be activated within a few milliseconds by single action potentials. The number of vesicles that release their contents in response to an action potential depends on the resting presynaptic membrane potential and on the calcium concentration [105]. Synaptic serotonin release also displays short-term plasticity phenomena in response to repetitive activity, such as paired-pulse facilitation, which occurs without changes in the amplitude of the presynaptic calcium currents [106], consistent with the residual calcium hypothesis [107]. Synaptic depression also occurs when trains of several action potentials are produced in Retzius neurons. The balance between facilitation and depression depends on the stimulation frequency [108], which ultimately determines the total amount of neurotransmitter released in response to this type of electrical activity.

Since the postsynaptic receptors are located only a few nanometers away from the presynaptic release sites, synaptic release produces fast responses that are initiated with a very short delay (of a few milliseconds) and have a duration in the order of tenths of milliseconds [105]. Thus, postsynaptic responses are always synchronized with and convey immediate information about the presynaptic electrical activity. In addition, the small amount of transmitter released by the presynaptic terminals produces only local effects, restricted to postsynaptic terminals of target neurons. The reuptake of serotonin by presynaptic transporters also contributes to the short duration of postsynaptic responses [109].

In addition to small clear vesicles, the presynaptic terminals of serotonergic Retzius neurons contain serotonin in large dense core vesicles [110,111]. The serotonin content of these vesicles is 17 times that of small clear vesicles [111]. Dense core vesicles release their contents in the area surrounding the active zone at presynaptic terminals, in response to pairs or trains of stimuli at high frequencies [112], and add a slow component to the postsynaptic response [113].

#### Autoregulation of Serotonin Release at Presynaptic Terminals

**Figure 3:**
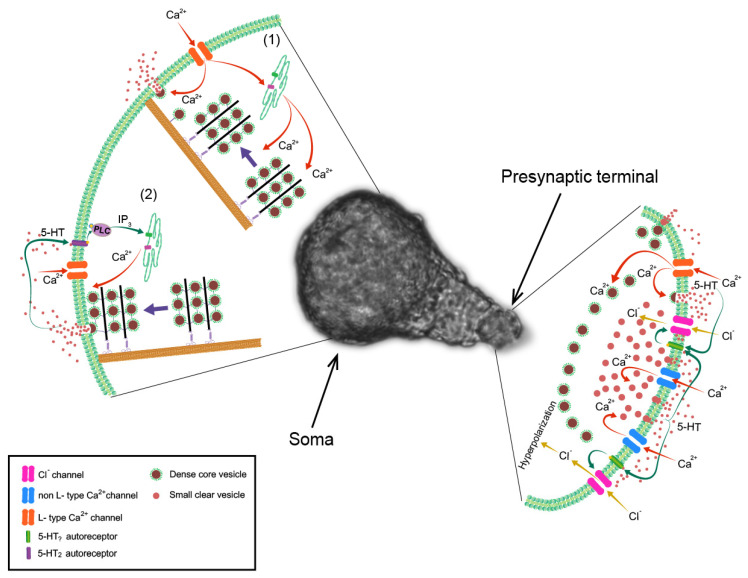
Autoregulation mechanisms in serotonergic Retzius neurons of the leech. A light-field micrography of a Retzius neuron in culture is shown in the middle. In presynaptic terminals, formed at the tip of the axonal stump (**right**), calcium (Ca^2+^) entry triggers serotonin release. Serotonin activates an autoreceptor coupled to chloride (Cl^−^) channels that hyperpolarize the membrane, reduce the input resistance and decrease the excitability, thus immediately reducing subsequent serotonin release. In the soma (**left**), stimulation with trains of impulses initiates the mobilization of distant vesicles to the membrane (1). Upon initial serotonin release, a 5-HT_2_ autoreceptor stimulates formation of IP_3_ through activation of PLC. IP_3_-dependent calcium release from the endoplasmic reticulum allows mobilized vesicles to fuse with the membrane (2), thus engaging a positive feedback loop that sustains serotonin release for minutes, even in the absence of further electrical activity.

Action potentials in cultured Retzius neurons end with a fast post-potential hyperpolarization, produced by outward potassium currents, which is followed by a slower hyperpolarization. Our research group found that this post-potential hyperpolarization is an autoregulatory response produced by serotonin released from the same neuron: it is blocked if secretion is inhibited by the absence of extracellular calcium, if the cell is depleted of serotonin by pre-incubation with reserpine or if serotonin antagonists are added to the extracellular solution [114]. This post-potential response is reversed if the chloride (Cl^−^) transmembrane gradient is inverted by injection of Cl^−^ ions through an intracellular electrode. All this evidence has shown that serotonin released from Retzius neurons activates 5-HT autoreceptors that are coupled to Cl^−^ channels (Figure 3).

This autoinhibitory response can be recorded with an intracellular electrode placed in the soma of Retzius neurons, which have been isolated, keeping a stump of the primary axon, where presynaptic terminals are formed at the tip. As explained above, in these conditions, the cells are practically isopotential, so that small electrical events happening at the presynaptic terminals can be readily recorded in the soma. However, the autoinhibitory response is not observed in cells that were isolated, taking only the soma (**without** a segment of the axon), where presynaptic terminals are absent, nor in Retzius neurons in situ in the central nervous system ganglia, where presynaptic terminals are located at the tips of long axons, and are, thus, electrotonically distant from the soma. This evidence shows that the autoinhibitory response is uniquely localized at presynaptic terminals, but not at the soma or the primary axon of Retzius neurons [115]. This autoinhibitory mechanism thus acts like a presynaptic inhibition [116] mechanism, regulating neurotransmitter release at the terminal.

What is the effect of this autoinhibitory mechanism on presynaptic terminals? Since the Cl^−^ equilibrium potential (ECl^−^) is near the resting membrane potential, the hyperpolarization produced by activation of Cl^−^ channels is not very large. Nevertheless, the activation of this conductance in the membrane decreases its input resistance, making it more difficult to change its voltage. It also holds the membrane potential near ECl^−^, thus decreasing the excitability of the cell [114] (Figure 3). The specific localization of this mechanism to the presynaptic terminals allows a very local autoregulation of electrical activity within the terminals. Action potentials in these neurons are initiated in the axonal cone, near the soma, and are actively conducted along the axon. Due to the specific localization of the autoinhibitory mechanism to the presynaptic terminals, this mechanism does not alter the initiation or propagation of the action potentials, but it is expected to have a local effect when an action potential invades the presynaptic terminals. Although it would be experimentally difficult to study the local effect of the autoinhibitory mechanism in the terminals, mathematical modelling has allowed us to predict that an action potential invading the presynaptic terminal shortly after previous activity that caused serotonin release would be decreased in amplitude by the presence of the active chloride conductance [114], and thus, the amount of serotonin released in response to this action potential would be smaller. In this way, the release of serotonin in response to an action potential arriving at the presynaptic terminal decreases the release in response to immediately subsequent nerve impulses. This allows for continuous and precise regulation of the ongoing electrical activity arriving at the terminal and of the consequent release of serotonin, at an impulse-by-impulse timescale.

The peri-synaptic release of serotonin from dense core vesicles that occurs upon repetitive stimulation of Retzius neurons [110,112] also participates in the activation of the autoinhibitory responses in the terminals, contributing more when the neurons are stimulated at higher frequencies [115] (Figure 3).

The inhibitory autoregulation present at presynaptic terminals may have a functional role not only regulating the post-synaptic responses produced by serotonin release but also preventing the depletion of the presynaptic releasable vesicle pool, thus optimizing the function of the synapse. It also makes sense to have such an inhibitory mechanism at a site where transmitter release conveys information about presynaptic electrical activity: the postpotential hyperpolarization may stop transmitter release shortly after the action potential, contributing to shaping postsynaptic responses and keeping them synchronized to presynaptic action potentials.

### 3.2. Extrasynaptic Serotonin Release at Retzius Neurons

Serotonergic Retzius neurons were one of the first neurons in which the extrasynaptic release of neurotransmitters was demonstrated [5]. Extrasynaptic secretion has been proposed to produce paracrine modulation of neighboring or distant cells. This type of communication in the nervous system requires the diffusion of neurotransmitters in volumes of intercellular space that are much larger than the synaptic clefts (for this reason it has also been called “volume transmission” [117]), and thus, it relies on the exocytosis of large amounts of molecules necessary to reach distant targets in sufficient concentrations to activate their receptors.

Retzius neurons display extrasynaptic release of serotonin from the soma, as well as from the primary axon. It is noteworthy that, as described above, extrasynaptic serotonin release from the somatodendritic region of 5-HT neurons in the raphe nuclei of mammals was also shown later [3,118], so extrasynaptic release seems to be a conserved feature of serotonergic systems. Most of the known neurotransmitters have also been shown to be released extrasynaptically in various areas of the nervous system of vertebrates and invertebrates [50], but the detailed mechanisms regulating this release remain elusive. The large soma of Retzius neurons has allowed a detailed study of the mechanisms regulating somatic serotonin release.

The soma of Retzius neurons contains thousands of dense core vesicles [119], forming clusters, which rest at a distance of more than one micron from the plasma membrane [120], in evident contrast with presynaptic terminals, where the vesicles are docked at the active zone [110]. Somatic secretion thus requires these vesicles to be mobilized over long distances towards the plasma membrane, which requires the entry of a large amount of calcium and its diffusion in the cytoplasm. This is only achieved by repetitive electrical activity at high frequencies [5], in contrast to synaptic secretion, which occurs in response to single action potentials [105]. High-frequency stimulation produces activation of L-type calcium channels in the soma [5]. Since these channels show little or no inactivation, they allow a larger flux of calcium ions into the cytoplasm than those in presynaptic terminals, which are generally of N, T, or P/Q types [121] and rapidly inactivate. In addition, the soma has large cisternae of endoplasmic reticulum (ER), where cytoplasmic calcium activates ryanodine receptors, thus producing calcium-induced calcium release [120]. The release of calcium from the ER amplifies the calcium signal in the cytoplasm and facilitates its propagation, allowing calcium to reach the vesicles in their resting sites, distant from the plasma membrane, and to trigger their mobilization [4]. The vesicles are slowly mobilized towards the plasma membrane by motor proteins, such as kinesin or dynein, that move along microtubules connecting the vesicle distant resting sites with the plasma membrane [122]. Vesicles do not arrive at the membrane all at once, but sequentially, a few vesicles at a time.

In addition to its role in vesicle mobilization, calcium is also essential for triggering the fusion of vesicles with the plasma membrane to produce exocytosis of neurotransmitters. In the soma of Retzius neurons the calcium that enters the cytoplasm during a short burst of electrical stimulation can produce the fusion of a few vesicles that are near the membrane. However, by the time most of the vesicles mobilized from more internal sites arrive at the membrane, the calcium signal produced by that short burst of electrical activity has already decayed. Nevertheless, in these neurons, a brief train of ten action potentials at frequencies of 10–20 Hz, lasting only half a second to a second, is able to produce secretion lasting for several minutes. How is the fusion of the vesicles that slowly arrive at the membrane produced? An autoregulatory mechanism producing positive feedback is crucial for ensuring that all the mobilized vesicles fuse with the membrane and release their contents.

#### Positive Autoregulation of Serotonin Release from the Soma

In Retzius neurons, vesicles resting near the plasma membrane can fuse with it in response to the calcium that enters the cytoplasm during or shortly after electrical activity. These vesicles release their serotonin contents to the surrounding environment, where the transmitter can diffuse and reach other neurons, but importantly, it also activates 5-HT_2_ autoreceptors located on the soma membrane. These autoreceptors are coupled through G proteins to PLC, which, as explained above, breaks down PIP_2_ into IP_3_ and DAG. IP_3_ can, in turn, activate its receptors in the membrane of ER cisternae near the plasma membrane and trigger a local release of calcium from these intracellular stores. This produces a localized submembrane increase in calcium concentration, which is enough to produce the fusion of the vesicles that subsequently arrive at the membrane. Serotonin released from these newly arriving vesicles, in turn, activates the autoreceptors again, triggering more calcium release from the ER near the membrane and allowing the fusion of the vesicles that come after them [4]. In this way, a positive feedback loop is established between serotonin exocytosis and intracellular calcium release (Figure 3). This feedback enables the fusion of all the vesicles whose mobilization was triggered by the calcium signal produced during previous electrical stimulation. However, since the calcium signal during this feedback is only localized to the sub-membrane region, it does not trigger the mobilization of more vesicles, thus avoiding the depletion of secretory vesicles in the cell. Therefore, the number of vesicles that are mobilized is only determined by electrical activity, whereas the subsequent feedback loop ensures that all the mobilized vesicles are fused with the plasma membrane and release their contents.

With this positive feedback, Retzius neurons are able to release the contents of thousands of vesicles during several minutes in response to only a brief train of electrical activity, while preserving vesicles to release upon further stimulation. This is also an efficient mechanism that allows exocytosis to continue for a long time without sustaining high-frequency electrical activity and continuous calcium entry, which would be toxic for the cell [123].

In addition to the autoregulatory effects mediated by autoreceptors, the reuptake of serotonin into 5-HT neurons through the active serotonin reuptake transporter contributes to shaping pre- and postsynaptic responses to serotonin. The experimental advantages that Retzius neurons offer have allowed for showing that SERT moves serotonin along with sodium (Na^+^) ions. The reuptake of each 5-HT molecule involves the influx of at least two Na^+^ ions, generating an inward Na^+^ current in 5-HT neurons as soon as serotonin is released in response to each action potential [109]. Serotonin reuptake occurs both in the soma and in the axon of Retzius neurons, and thus, it can presumably modulate electrical activity and shape the autoregulatory effects of serotonin in both neuronal compartments. In addition, the reuptake of serotonin by SERT decreases the amplitude and duration of postsynaptic responses to serotonin. It is, however, not clear how serotonin reuptake affects somatic release and the autoregulatory mechanism activated by serotonin in the soma of these neurons.

### 3.3. Possible Functions of the Different Autoregulation Mechanisms at Different Release Sites of the Neuron

As shown above, the presynaptic terminals and the soma of serotonergic Retzius neurons have different autoregulatory mechanisms that have opposite effects: while the autoregulation in presynaptic terminals stops or limits subsequent transmitter release, the mechanism in the soma promotes further release of serotonin after electrical activity ceases. The markedly different autoregulation mechanisms shown at different compartments of the same neuron show the specific and differential localization of different 5-HT autoreceptor types at each of these neuronal compartments, activating a different intracellular response in each of them. This difference in autoregulatory mechanisms is relevant in the context of the mechanisms of release at each structure, as well as in the context of the functions that each type of release has in the nervous system.

Synaptic transmission is a very specialized form of communication that conveys information about the ongoing activity of the presynaptic neuron. The active zone, with its high density of voltage-gated calcium channels and a docked pool of readily releasable vesicles, allows transmitter release in milliseconds following each action potential that invades the presynaptic terminal. Due to the short distance and restricted volume of the synaptic cleft, the postsynaptic response also occurs on the millisecond timescale, and the small amount of transmitter released at synapses only allows the activation of receptors localized on the postsynaptic terminal facing the release site. The transmitter is also rapidly removed from the synaptic cleft, either by presynaptic or glial transporters, or by degrading enzymes (depending on the neurotransmitter), so the response is also very short-lived. In this context, an autoregulatory mechanism that stops or restricts subsequent transmitter release after each action potential contributes to maintaining the fine temporal resolution of the postsynaptic responses, which is linked to presynaptic activity. At this level, even small changes in the amount of release are significant, since the amount of transmitter released by synaptic terminals is small. Furthermore, since the presynaptic terminals of serotonergic Retzius neurons display both synaptic facilitation and synaptic depression, there are certain frequencies at which electrical activity is most effective to trigger transmission. It is, thus, possible that this autoregulatory mechanism aids the terminal in selecting the frequencies that are optimal for transmitter release, while avoiding higher frequencies that produce synaptic depression.

The specific localization of these inhibitory autoreceptors to the presynaptic terminals, and not the soma or primary axon, allows a very localized regulation of the electrical activity at presynaptic terminals, without affecting the integration of synaptic inputs that the 5-HT neuron receives, which occurs in the axonal cone, where the axon begins.

On the other hand, extrasynaptic communication is thought to set a generalized and sustained “basal state” or “tone” on neuronal populations or circuits. In this case, transmitters must diffuse in large volumes of intercellular fluid [117] to reach distant and numerous target neurons in concentrations sufficient to activate their receptors. This requires the release of very large amounts of neurotransmitter, in what has been called “volume transmission”. The mechanisms underlying somatic secretion in Retzius neurons enable the neuron to release these large amounts of serotonin, since thousands of dense core vesicles release their contents during this process, even when the cell is only activated for a short period of time. In addition, the slow time course of this type of release may regulate slow processes in the nervous system that are not easily explainable by fast synaptic transmission, such as the modulation of feeding or social behavior. Although this type of secretion has not been studied with such detail in mammals, similar processes may modulate higher functions, such as attention or mood, in humans. In this context, the autoregulatory mechanism present at the soma of these neurons, which activates a positive feedback that sustains serotonin secretion, seems ideal for the functions of extrasynaptic communication, since it ensures the slow and long-lasting release of neurotransmitter over long periods, even in the absence of sustained electrical activity, provided that a brief high-frequency activation occurred. In this respect, extrasynaptic release does not convey information about the ongoing activity of the neuron, but it contributes to setting different basal states in the circuits depending on the type of electrical activity that the releasing neuron displays.

In summary, serotonergic Retzius neurons, with their large size and the possibility of being isolated and kept in culture, have been a model neuron to study the automodulatory mechanisms involved in regulating compartmentalized serotonin release from presynaptic terminals and from the soma of these neurons. In these neurons serotonin release from presynaptic terminals upon single action potentials produces a local autoinhibition of electrical activity by activating autoreceptors coupled to chloride channels, which decrease the input resistance and subsequent excitability, thus decreasing the release of serotonin produced by subsequently incoming action potentials. On the other hand, serotonin released from the soma after repetitive high-frequency electrical activity engages in a positive feedback loop that sustains somatic serotonin secretion for long times by activating autoreceptors coupled to phospholipase C, which trigger IP_3_-induced calcium release from intracellular stores near the membrane, thus providing calcium to sustain exocytosis long after electrical activity ceased.

## 4. Conclusions and Future Directions

In addition to its modulatory actions on other neuron types, serotonin exerts autoregulatory actions back onto 5-HT neurons, regulating their electrical activity and its own release. This occurs in both invertebrates and vertebrates, including mammals.

The study of autoregulation in mammals has yielded extensive information about the types of autoreceptors present in 5-HT neurons, their localization throughout the nervous system and some of their autoregulatory effects. However, the complexity of the mammalian nervous system has posed difficulties in the understanding of these processes, and the extensive literature on this subject provides numerous pieces of a puzzle that is not easy to put together. We have critically reviewed this information, trying to devise a clearer scheme for this complex subject. On the other hand, studies of 5-HT neurons of an invertebrate, the medicinal leech, have allowed a more straightforward study of the different actions that serotonin exerts on these neurons upon its release.

Depending on the type of autoreceptors activated, serotonin can inhibit or stimulate the electrical activity of 5-HT neurons and its own release. In mammals, different types of autoreceptors in the somatodendritic compartment of 5-HT neurons interact to finely tune the electrical activity of these neurons. In addition, axonal or terminal inhibitory autoreceptors of a different type are thought to regulate the conduction of electrical activity and serotonin release (this has, however, not been directly demonstrated). Studying the detailed mechanisms of autoregulation at different compartments of 5-HT neurons in the mammalian nervous system would give a more complete understanding of how serotonin release is modulated and relate its autoregulatory actions to specific functions of serotonin in each brain region. Studies in simpler nervous systems of invertebrates, however, have opened a window to the complexity of serotonergic autoregulation. In 5-HT neurons of the leech, inhibitory autoreceptors coupled to chloride channels, specifically located on presynaptic terminals, have been shown to regulate the excitability of the local membrane and regulate the release of serotonin from synapses. On the other hand, a different type of 5-HT autoreceptors in the soma engage in a positive feedback loop that sustains the release of large amounts of serotonin through the release of calcium from local intracellular stores. The experimental advantages of these neurons open the path to further study how serotonergic autoregulation modulates the firing frequency of 5-HT neurons, the consequent changes in serotonin release, its interaction with serotonin reuptake, as well as other open questions. The specific—and opposite—autoregulatory actions of serotonin at each of these compartments of the same neuron are aligned with the different release modalities acting in each of them, as well as with the effects of each of these types of release. In this way, serotonergic autoregulation contributes to the compartmentalization of serotonin release and to the multimodality of serotonergic neurons, which regulate a myriad of physiological processes, despite their small number.

## Data Availability

No new data were created or analyzed in this study. Data sharing is not applicable to this article.

## Abbreviations

The following abbreviations are used in this manuscript:

5-HT	5-Hydroxytryptamine, Serotonin
DA	Dopamine
DAG	Diacylglycerol
DRN	Dorsal Raphe Nucleus
ECl^−^	Chloride Equilibrium Potential
ER	Enddoplasmic Reticulum
GIRK	G protein-Coupled Inwardly-Rectifying Potassium Channel
IP_3_	Inositol Tripshosphate
MDMA	3,4-methylenedioxymethamphetamine
PIP_2_	Phosphatydyl Inositol Bisphosphate
PKC	Protein Kinase C
PLC	Phospholipase C
SSRI	Selective Serotonin Reuptake Inhibitor
VTA	Ventral Tegmental Area

## References

[1] Underwood M.D., Khaibulina A.A., Ellis S.P., Moran A., Rice P.M., Mann J.J., Arango V. (1999). Morphometry of the Dorsal Raphe Nucleus erotonergic Neurons in Suicide Victims. Biol. Psychiatry.

[2] Trueta C., G. Cercós M., Fatima-Shad K. (2024). Serotonin in the Nervous System: Few Neurons Regulating Many Functions. Serotonin—Neurotransmitter and Hormone of Brain, Bowels and Blood.

[3] Kaushalya S.K., Desai R., Arumugam S., Ghosh H., Balaji J., Maiti S. (2008). Three-Photon Microscopy Shows That Somatic Release Can Be a Quantitatively Significant Component of Serotonergic Neurotransmission in the Mammalian Brain. J. Neurosci. Res.

[4] Leon-Pinzon C., Cercós M.G., Noguez P., Trueta C., De-Miguel F.F. (2014). Exocytosis of Serotonin from the Neuronal Soma Is Sustained by a Serotonin and Calcium-Dependent Feedback Loop. Front. Cell. Neurosci..

[5] Trueta C., Méndez B., De-Miguel F.F. (2003). Somatic Exocytosis of Serotonin Mediated by L-Type Calcium Channels in Cultured Leech Neurones. J. Physiol. (Lond.).

[6] Chazal G., Ralston H.J. (1987). Serotonin-Containing Structures in the Nucleus Raphe Dorsalis of the Cat: An Ultrastructural Analysis of Dendrites, Presynaptic Dendrites, and Axon Terminals. J. Comp. Neurol..

[7] Dahlström A., Fuxe K. (1964). Localization of Monoamines in the Lower Brain Stem. Experientia.

[8] Aghajanian G.K. (1972). Chemical-Feedback Regulation of Serotonin-Containing Neurons in Brain. Ann. N. Y. Acad. Sci..

[9] Gallager D.W., Aghajanian G.K. (1976). Inhibition of Firing of Raphe Neurones by Tryptophan and 5-Hydroxytryptophan: Blockade by Inhibiting Serotonin Synthesis with Ro-4-4602. Neuropharmacology.

[10] Trulson M.E., Jacobs B.L. (1976). Dose-Response Relationships between Systemically Administered L-Tryptophan or L-5-Hydroxytryptophan and Raphe Unit Activity in the Rat. Neuropharmacology.

[11] Sprouse J.S., Aghajanian G.K. (1987). Electrophysiological Responses of Serotoninergic Dorsal Raphe Neurons to 5-HT1A and 5-HT1B Agonists. Synapse.

[12] Rogawski M.A., Aghajanian G.K. (1981). Serotonin Autoreceptors on Dorsal Raphe Neurons: Structure-Activity Relationships of Tryptamine Analogs. J. Neurosci..

[13] Gobert A., Lejeune F., Rivet J.M., Audinot V., Newman-Tancredi A., Millan M.J. (1995). Modulation of the Activity of Central Serotoninergic Neurons by Novel serotonin1A Receptor Agonists and Antagonists: A Comparison to Adrenergic and Dopaminergic Neurons in Rats. J. Pharmacol. Exp. Ther..

[14] Fornal C.A., Litto W.J., Metzler C.W., Marrosu F., Tada K., Jacobs B.L. (1994). Single-Unit Responses of Serotonergic Dorsal Raphe Neurons to 5-HT1A Agonist and Antagonist Drug Administration in Behaving Cats. J. Pharmacol. Exp. Ther..

[15] de Montigny C., Aghajanian G.K. (1977). Preferential Action of 5-Methoxytryptamine and 5-Methoxydimethyltryptamine on Presynaptic Serotonin Receptors: A Comparative Iontophoretic Study with LSD and Serotonin. Neuropharmacology.

[16] VanderMaelen C.P., Matheson G.K., Wilderman R.C., Patterson L.A. (1986). Inhibition of Serotonergic Dorsal Raphe Neurons by Systemic and Iontophoretic Administration of Buspirone, a Non-Benzodiazepine Anxiolytic Drug. Eur. J. Pharmacol..

[17] Aghajanian G.K., Lakoski J.M. (1984). Hyperpolarization of Serotonergic Neurons by Serotonin and LSD: Studies in Brain Slices Showing Increased K+ Conductance. Brain Res..

[18] Hjorth S., Magnusson T. (1988). The 5-HT 1A Receptor Agonist, 8-OH-DPAT, Preferentially Activates Cell Body 5-HT Autoreceptors in Rat Brain in Vivo. Naunyn Schmiedebergs Arch. Pharmacol..

[19] Invernizzi R., Carli M., Di Clemente A., Samanin R. (1991). Administration of 8-Hydroxy-2-(Di- *n* -Propylamino)Tetralin in Raphe Nuclei Dorsalis and Medianus Reduces Serotonin Synthesis in the Rat Brain: Differences in Potency and Regional Sensitivity. J. Neurochem..

[20] Sharp T., Bramwell S.R., Clark D., Grahame-Smith D.G. (1989). In Vivo Measurement of Extracellular 5-Hydroxytryptamine in Hippocampus of the Anaesthetized Rat Using Microdialysis: Changes in Relation to 5-Hydroxytryptaminergic Neuronal Activity. J. Neurochem..

[21] Bonvento G., Scatton B., Claustre Y., Rouquier L. (1992). Effect of Local Injection of 8-OH-DPAT into the Dorsal or Median Raphe Nuclei on Extracellular Levels of Serotonin in Serotonergic Projection Areas in the Rat Brain. Neurosci. Lett..

[22] Wang R.Y., Aghajanian G.K. (1977). Antidromically Identified Serotonergic Neurons in the Rat Midbrain Raphe: Evidence for Collateral Inhibition. Brain Res.

[23] Wang R.Y., Aghajanian G.K. (1978). Collateral Inhibition of Serotonergic Neurones in the Rat Dorsal Raphe Nucleus: Pharmacological Evidence. Neuropharmacology.

[24] Pan Z.Z., Colmers W.F., Williams J.T. (1989). 5-HT-Mediated Synaptic Potentials in the Dorsal Raphe Nucleus: Interactions with Excitatory Amino Acid and GABA Neurotransmission. J. Neurophysiol..

[25] Moulédous L., Roullet P., Guiard B.P., Guiard B.P., Di Giovanni G. (2018). Brain Circuits Regulated by the 5-HT2A Receptor: Behavioural Consequences on Anxiety and Fear Memory. 5-HT2A Receptors in the Central Nervous System.

[26] Sari Y., Miquel M.C., Brisorgueil M.J., Ruiz G., Doucet E., Hamon M., Vergé D. (1999). Cellular and Subcellular Localization of 5-hydroxytryptamine1B Receptors in the Rat Central Nervous System: Immunocytochemical, Autoradiographic and Lesion Studies. Neuroscience.

[27] Sari Y. (2004). Serotonin1B Receptors: From Protein to Physiological Function and Behavior. Neurosci. Biobehav. Rev..

[28] Riad M., Garcia S., Watkins K.C., Jodoin N., Doucet E., Langlois X., el Mestikawy S., Hamon M., Descarries L. (2000). Somatodendritic Localization of 5-HT1A and Preterminal Axonal Localization of 5-HT1B Serotonin Receptors in Adult Rat Brain. J. Comp. Neurol.

[29] Kia H.K., Miquel M.C., Brisorgueil M.J., Daval G., Riad M., El Mestikawy S., Hamon M., Vergé D. (1996). Immunocytochemical Localization of serotonin1A Receptors in the Rat Central Nervous System. J. Comp. Neurol..

[30] Sotelo C., Cholley B., El Mestikawy S., Gozlan H., Hamon M. (1990). Direct Immunohistochemical Evidence of the Existence of 5-HT_1A_ Autoreceptors on Serotoninergic Neurons in the Midbrain Raphe Nuclei. Eur. J. Neurosci..

[31] Verge D., Daval G., Patey A., Gozlan H., el Mestikawy S., Hamon M. (1985). Presynaptic 5-HT Autoreceptors on 5-HT Cell Bodies and/or Dendrites but Not Terminals Are of the 5-HT1A Subtype. Eur. J. Pharmacol..

[32] Boschert U., Amara D.A., Segu L., Hen R. (1994). The Mouse 5-hydroxytryptamine1B Receptor Is Localized Predominantly on Axon Terminals. Neuroscience.

[33] Bidmon H.J., Schleicher A., Wicke K., Gross G., Zilles K. (2001). Localisation of mRNA for H5-HT1B and H5-HT1D Receptors in Human Dorsal Raphe. Naunyn Schmiedebergs Arch. Pharmacol..

[34] Diaz S.L., Doly S., Narboux-Nême N., Fernández S., Mazot P., Banas S.M., Boutourlinsky K., Moutkine I., Belmer A., Roumier A. (2012). 5-HT(2B) Receptors Are Required for Serotonin-Selective Antidepressant Actions. Mol. Psychiatry.

[35] Corradetti R., Le Poul E., Laaris N., Hamon M., Lanfumey L. (1996). Electrophysiological Effects of N-(2-(4-(2-Methoxyphenyl)-1-Piperazinyl)Ethyl)-N-(2-Pyridinyl) Cyclohexane Carboxamide (WAY 100635) on Dorsal Raphe Serotonergic Neurons and CA1 Hippocampal Pyramidal Cells in Vitro. J. Pharmacol. Exp. Ther..

[36] Craven R., Grahame-Smith D., Newberry N. (1994). WAY-100635 and GR127935: Effects on 5-Hydroxytryptamine-Containing Neurones. Eur. J. Pharmacol..

[37] Fletcher A., Forster E.A., Bill D.J., Brown G., Cliffe I.A., Hartley J.E., Jones D.E., McLenachan A., Stanhope K.J., Critchley D.J.P. (1995). Electrophysiological, Biochemical, Neurohormonal and Behavioural Studies with WAY-100635, a Potent, Selective and Silent 5-HT1A Receptor Antagonist. Behav. Brain Res..

[38] Johnson D.A., Gartside S.E., Ingram C.D. (2002). 5-HT1A Receptor-Mediated Autoinhibition Does Not Function at Physiological Firing Rates: Evidence from in Vitro Electrophysiological Studies in the Rat Dorsal Raphe Nucleus. Neuropharmacology.

[39] Fornal C.A., Metzler C.W., Gallegos R.A., Veasey S.C., McCreary A.C., Jacobs B.L. (1996). WAY-100635, a Potent and Selective 5-hydroxytryptamine1A Antagonist, Increases Serotonergic Neuronal Activity in Behaving Cats: Comparison with (S)-WAY-100135. J. Pharmacol. Exp. Ther..

[40] Andrade R., Huereca D., Lyons J.G., Andrade E.M., McGregor K.M. (2015). 5-HT1A Receptor-Mediated Autoinhibition and the Control of Serotonergic Cell Firing. ACS Chem. Neurosci..

[41] Courtney N.A., Ford C.P. (2016). Mechanisms of 5-HT_1A_ Receptor-mediated Transmission in Dorsal Raphe Serotonin Neurons. J. Physiol..

[42] Bayliss D.A., Li Y.W., Talley E.M. (1997). Effects of Serotonin on Caudal Raphe Neurons: Activation of an Inwardly Rectifying Potassium Conductance. J. Neurophysiol..

[43] Katayama J., Yakushiji T., Akaike N. (1997). Characterization of the K^+^ Current Mediated by 5-HT1A Receptor in the Acutely Dissociated Rat Dorsal Raphe Neurons. Brain Res..

[44] Sharp T., Bramwell S.R., Grahame-Smith D.G. (1989). 5-HT1 Agonists Reduce 5-Hydroxytryptamine Release in Rat Hippocampus in Vivo as Determined by Brain Microdialysis. Br. J. Pharmacol..

[45] Andrade R., Nicoll R.A. (1987). Pharmacologically Distinct Actions of Serotonin on Single Pyramidal Neurones of the Rat Hippocampus Recorded in Vitro. J. Physiol..

[46] Ropert N. (1988). Inhibitory Action of Serotonin in CA1 Hippocampal Neurons in Vitro. Neuroscience.

[47] Richardson-Jones J.W., Craige C.P., Guiard B.P., Stephen A., Metzger K.L., Kung H.F., Gardier A.M., Dranovsky A., David D.J., Beck S.G. (2010). 5-HT1A Autoreceptor Levels Determine Vulnerability to Stress and Response to Antidepressants. Neuron.

[48] Blier P., Piñeyro G., el Mansari M., Bergeron R., de Montigny C. (1998). Role of Somatodendritic 5-HT Autoreceptors in Modulating 5-HT Neurotransmission. Ann. N. Y. Acad. Sci..

[49] Doucet E., Pohl M., Fattaccini C.M., Adrien J., Mestikawy S.E., Hamon M. (1995). In Situ Hybridization Evidence for the Synthesis of 5-HT1B Receptor in Serotoninergic Neurons of Anterior Raphe Nuclei in the Rat Brain. Synapse.

[50] Trueta C., De-Miguel F.F. (2012). Extrasynaptic Exocytosis and Its Mechanisms: A Source of Molecules Mediating Volume Transmission in the Nervous System. Front. Physiol..

[51] Bouhelal R., Smounya L., Bockaert J. (1988). 5-HT1B Receptors Are Negatively Coupled with Adenylate Cyclase in Rat Substantia Nigra. Eur. J. Pharmacol..

[52] Pullarkat S.R., Mysels D.J., Tan M., Cowen D.S. (1998). Coupling of Serotonin 5-HT1B Receptors to Activation of Mitogen-Activated Protein Kinase (ERK-2) and P70 S6 Kinase Signaling Systems. J. Neurochem..

[53] Le Grand B., Panissié A., Pauwels P.J., John G.W. (1998). Activation of Recombinant h 5-HT1B and h 5-HT1D Receptors Stably Expressed in C6 Glioma Cells Produces Increases in Ca^2+^-Dependent K^+^ Current. Naunyn Schmiedebergs Arch. Pharmacol..

[54] Andrade R., Malenka R.C., Nicoll R.A. (1986). A G Protein Couples Serotonin and GABAB Receptors to the Same Channels in Hippocampus. Science.

[55] Gadgaard C., Jensen A.A. (2020). Functional Characterization of 5-HT1A and 5-HT1B Serotonin Receptor Signaling through G-Protein-Activated Inwardly Rectifying K^+^ Channels in a Fluorescence-Based Membrane Potential Assay. Biochem. Pharmacol..

[56] Mizutani H., Hori T., Takahashi T. (2006). 5-HT1B Receptor-Mediated Presynaptic Inhibition at the Calyx of Held of Immature Rats. Eur. J. Neurosci..

[57] Maura G., Roccatagliata E., Raiteri M. (1986). Serotonin Autoreceptor in Rat Hippocampus: Pharmacological Characterization as a Subtype of the 5-HT1 Receptor. Naunyn-Schmiedeberg’s Arch. Pharmacol..

[58] Hjorth S., Tao R. (1991). The Putative 5-HT1B Receptor Agonist CP-93,129 Suppresses Rat Hippocampal 5-HT Release in Vivo: Comparison with RU 24969. Eur. J. Pharmacol..

[59] Sayer T.J.O., Hannon S.D., Redfern P.H., Martin K.F. (1999). Diurnal Variation in 5-HT_1B_ Autoreceptor Function in the Anterior Hypothalamus *in Vivo*: Effect of Chronic Antidepressant Drug Treatment. Br. J Pharmacol..

[60] Hertel P., Nomikos G.G., Svensson T.H. (1999). The Antipsychotic Drug Risperidone Interacts with Auto- and Hetero-Receptors Regulating Serotonin Output in the Rat Frontal Cortex. Neuropharmacology.

[61] Adell A., Celada P., Artigas F. (2001). The Role of 5-HT1B Receptors in the Regulation of Serotonin Cell Firing and Release in the Rat Brain. J. Neurochem..

[62] Knobelman D.A., Kung H.F., Lucki I. (2000). Regulation of Extracellular Concentrations of 5-Hydroxytryptamine (5-HT) in Mouse Striatum by 5-HT(1A) and 5-HT(1B) Receptors. J. Pharmacol. Exp. Ther..

[63] Schmidt A.W., Macor J.E., Schultz D.W. (1990). CP-93,129: A Potent and Selective 5-HT1B Receptor Agonist. Soc. Neurosci. Meet. Abstr..

[64] Daws L.C., Gould G.G., Teicher S.D., Gerhardt G.A., Frazer A. (2000). 5-HT(1B) Receptor-Mediated Regulation of Serotonin Clearance in Rat Hippocampus in Vivo. J. Neurochem..

[65] Hagan C.E., McDevitt R.A., Liu Y., Furay A.R., Neumaier J.F. (2012). 5-HT(1B) Autoreceptor Regulation of Serotonin Transporter Activity in Synaptosomes. Synapse.

[66] Montañez S., Munn J.L., Owens W.A., Horton R.E., Daws L.C. (2014). 5-HT1B Receptor Modulation of the Serotonin Transporter in Vivo: Studies Using KO Mice. Neurochem. Int..

[67] Evrard A., Laporte A.M., Chastanet M., Hen R., Hamon M., Adrien J. (1999). 5-HT1A and 5-HT1B Receptors Control the Firing of Serotoninergic Neurons in the Dorsal Raphe Nucleus of the Mouse: Studies in 5-HT1B Knock-out Mice. Eur. J. Neurosci..

[68] Engel G., Göthert M., Hoyer D., Schlicker E., Hillenbrand K. (1986). Identity of Inhibitory Presynaptic 5-Hydroxytryptamine (5-HT) Autoreceptors in the Rat Brain Cortex with 5-HT1B Binding Sites. Naunyn Schmiedebergs Arch. Pharmacol..

[69] Göthert M. (1990). Presynaptic Serotonin Receptors in the Central Nervous System. Ann. N. Y. Acad. Sci..

[70] Tanaka E., North R.A. (1993). Actions of 5-Hydroxytryptamine on Neurons of the Rat Cingulate Cortex. J. Neurophysiol..

[71] Sarhan H., Fillion G. (1999). Differential Sensitivity of 5-HT1B Auto and Heteroreceptors. Naunyn Schmiedebergs Arch. Pharmacol..

[72] Cassel J.C., Jeltsch H. (1995). Serotonergic Modulation of Cholinergic Function in the Central Nervous System: Cognitive Implications. Neuroscience.

[73] Maura G., Raiteri M. (1986). Cholinergic Terminals in Rat Hippocampus Possess 5-HT1B Receptors Mediating Inhibition of Acetylcholine Release. Eur. J. Pharmacol..

[74] Huang C., Yeh C., Wu M., Hsu K. (2013). A Single in Vivo Cocaine Administration Impairs 5-HT1B Receptor-induced Long-term Depression in the Nucleus Accumbens. J. Neurochem..

[75] Bagdy E., Kiraly I., Harsing L.G. (2000). Reciprocal Innervation between Serotonergic and GABAergic Neurons in Raphe Nuclei of the Rat. Neurochem. Res..

[76] Raymond J.R., Mukhin Y.V., Gelasco A., Turner J., Collinsworth G., Gettys T.W., Grewal J.S., Garnovskaya M.N. (2001). Multiplicity of Mechanisms of Serotonin Receptor Signal Transduction. Pharmacol. Ther..

[77] Roepke T.A., Smith A.W., Rønnekleiv O.K., Kelly M.J. (2012). Serotonin 5-HT2C Receptor-Mediated Inhibition of the M-Current in Hypothalamic POMC Neurons. Am. J. Physiol. Endocrinol. Metab..

[78] Belmer A., Quentin E., Diaz S.L., Guiard B.P., Fernandez S.P., Doly S., Banas S.M., Pitychoutis P.M., Moutkine I., Muzerelle A. (2018). Positive Regulation of Raphe Serotonin Neurons by Serotonin 2B Receptors. Neuropsychopharmacology.

[79] Doly S., Valjent E., Setola V., Callebert J., Hervé D., Launay J.-M., Maroteaux L. (2008). Serotonin 5-HT_2B_ Receptors Are Required for 3,4-Methylenedioxymethamphetamine-Induced Hyperlocomotion and 5-HT Release In Vivo and In Vitro. J. Neurosci..

[80] Craven R.M., Grahame-Smith D.G., Newberry N.R. (2001). 5-HT1A and 5-HT2 Receptors Differentially Regulate the Excitability of 5-HT-Containing Neurones of the Guinea Pig Dorsal Raphe Nucleus in Vitro. Brain Res..

[81] Benhadda A., Delhaye C., Moutkine I., Marques X., Russeau M., Le Magueresse C., Roumier A., Lévi S., Maroteaux L. (2023). 5-HT1A and 5-HT2B Receptor Interaction and Co-Clustering Regulate Serotonergic Neuron Excitability. iScience.

[82] Launay J., Schneider B., Loric S., Prada M.D., Kellermann O. (2006). Serotonin Transport and Serotonin Transporter-mediated Antidepressant Recognition Are Controlled by 5-HT_2B_ Receptor Signaling in Serotonergic Neuronal Cells. FASEB J..

[83] Diaz S.L., Maroteaux L. (2011). Implication of 5-HT(2B) Receptors in the Serotonin Syndrome. Neuropharmacology.

[84] Doly S., Bertran-Gonzalez J., Callebert J., Bruneau A., Banas S.M., Belmer A., Boutourlinsky K., Hervé D., Launay J.-M., Maroteaux L. (2009). Role of Serotonin via 5-HT2B Receptors in the Reinforcing Effects of MDMA in Mice. PLoS ONE.

[85] Descarries L., Watkins K.C., Garcia S., Beaudet A. (1982). The Serotonin Neurons in Nucleus Raphe Dorsalis of Adult Rat: A Light and Electron Microscope Radioautographic Study. J. Comp. Neurol..

[86] Chazal G., Ohara P.T. (1986). Vesicle-Containing Dendrites in the Nucleus Raphe Dorsalis of the Cat. A Serial Section Electron Microscopic Analysis. J. Neurocytol..

[87] Bunin M.A., Prioleau C., Mailman R.B., Wightman R.M. (1998). Release and Uptake Rates of 5-Hydroxytryptamine in the Dorsal Raphe and Substantia Nigra Reticulata of the Rat Brain. J. Neurochem..

[88] Bunin M.A., Wightman R.M. (1998). Quantitative Evaluation of 5-Hydroxytryptamine (Serotonin) Neuronal Release and Uptake: An Investigation of Extrasynaptic Transmission. J. Neurosci..

[89] O’Connor J.J., Kruk Z.L. (1991). Frequency Dependence of 5-HT Autoreceptor Function in Rat Dorsal Raphe and Suprachiasmatic Nuclei Studied Using Fast Cyclic Voltammetry. Brain Res..

[90] Stamford J.A., Davidson C., McLaughlin D.P., Hopwood S.E. (2000). Control of Dorsal Raphé 5-HT Function by Multiple 5-HT(1) Autoreceptors: Parallel Purposes or Pointless Plurality?. Trends Neurosci..

[91] de Kock C.P.J., Cornelisse L.N., Burnashev N., Lodder J.C., Timmerman A.J., Couey J.J., Mansvelder H.D., Brussaard A.B. (2006). NMDA Receptors Trigger Neurosecretion of 5-HT within Dorsal Raphe Nucleus of the Rat in the Absence of Action Potential Firing. J. Physiol. (Lond.).

[92] Colgan L.A., Putzier I., Levitan E.S. (2009). Activity-Dependent Vesicular Monoamine Transporter-Mediated Depletion of the Nucleus Supports Somatic Release by Serotonin Neurons. J. Neurosci..

[93] Colgan L.A., Cavolo S.L., Commons K.G., Levitan E.S. (2012). Action Potential-Independent and Pharmacologically Unique Vesicular Serotonin Release from Dendrites. J. Neurosci..

[94] Sukiasyan N., Hultborn H., Zhang M. (2009). Distribution of Calcium Channel Ca(V)1.3 Immunoreactivity in the Rat Spinal Cord and Brain Stem. Neuroscience.

[95] Lipscombe D., Helton T.D., Xu W. (2004). L-Type Calcium Channels: The Low Down. J. Neurophysiol..

[96] Unett D.J., Gatlin J., Anthony T.L., Buzard D.J., Chang S., Chen C., Chen X., Dang H.T.-M., Frazer J., Le M.K. (2013). Kinetics of 5-HT2B Receptor Signaling: Profound Agonist-Dependent Effects on Signaling Onset and Duration. J. Pharmacol. Exp. Ther..

[97] Davidson C., Stamford J.A. (1995). Evidence That 5-Hydroxytryptamine Release in Rat Dorsal Raphé Nucleus Is Controlled by 5-HT1A, 5-HT1B and 5-HT1D Autoreceptors. Br. J. Pharmacol..

[98] Azmitia E.C., Segal M. (1978). An Autoradiographic Analysis of the Differential Ascending Projections of the Dorsal and Median Raphe Nuclei in the Rat. J. Comp. Neurol..

[99] Hodgkin A.L., Huxley A.F. (1945). Resting and Action Potentials in Single Nerve Fibres. J. Physiol..

[100] Grashow R., Brookings T., Marder E. (2009). Reliable Neuromodulation from Circuits with Variable Underlying Structure. Proc. Natl. Acad. Sci. USA.

[101] Henderson L.P. (1983). The Role of 5-Hydroxytryptamine as a Transmitter between Identified Leech Neurones in Culture. J. Physiol. (Lond.).

[102] Nicholls J.G., Kuffler D.P. (1990). Quantal Release of Serotonin from Presynaptic Nerve Terminals. Neurochem. Int.

[103] Katz B. (2003). Neural Transmitter Release: From Quantal Secretion to Exocytosis and Beyond. J. Neurocytol..

[104] Henderson L.P., Kuffler D.P., Nicholls J., Zhang R. (1983). Structural and Functional Analysis of Synaptic Transmission between Identified Leech Neurones in Culture. J. Physiol. (Lond.).

[105] Dietzel I.D., Drapeau P., Nicholls J.G. (1986). Voltage Dependence of 5-Hydroxytryptamine Release at a Synapse between Identified Leech Neurones in Culture. J. Physiol..

[106] Stewart R.R., Adams W.B., Nicholls J.G. (1989). Presynaptic Calcium Currents and Facilitation of Serotonin Release at Synapses between Cultured Leech Neurones. J. Exp. Biol..

[107] Katz B., Miledi R. (1968). The Role of Calcium in Neuromuscular Facilitation. J. Physiol. (Lond.).

[108] Dittman J.S., Kreitzer A.C., Regehr W.G. (2000). Interplay between Facilitation, Depression, and Residual Calcium at Three Presynaptic Terminals. J. Neurosci..

[109] Bruns D., Engert F., Lux H.D. (1993). A Fast Activating Presynaptic Reuptake Current during Serotonergic Transmission in Identified Neurons of Hirudo. Neuron.

[110] Kuffler D.P., Nicholls J., Drapeau P. (1987). Transmitter Localization and Vesicle Turnover at a Serotoninergic Synapse between Identified Leech Neurons in Culture. J. Comp. Neurol..

[111] Bruns D., Riedel D., Klingauf J., Jahn R. (2000). Quantal Release of Serotonin. Neuron.

[112] Bruns D., Jahn R. (1995). Real-Time Measurement of Transmitter Release from Single Synaptic Vesicles. Nature.

[113] Trueta C. (2021). An Analytical Method to Measure the Contribution of Clear Synaptic and Dense-Core Peri-Synaptic Vesicles to Neurotransmitter Release from Synaptic Terminals with Two Classes of Secretory Vesicles. MethodsX.

[114] Cercós M.G., De-Miguel F.F., Trueta C. (2009). Real-Time Measurements of Synaptic Autoinhibition Produced by Serotonin Release in Cultured Leech Neurons. J. Neurophysiol..

[115] García-Ávila M., Torres X., Cercós M.G., Trueta C. (2021). Specific Localization of an Auto-Inhibition Mechanism at Presynaptic Terminals of Identified Serotonergic Neurons. Neuroscience.

[116] Bramley J.R., Sollars P.J., Pickard G.E., Dudek F.E. (2005). 5-HT1B Receptor-Mediated Presynaptic Inhibition of GABA Release in the Suprachiasmatic Nucleus. J. Neurophysiol..

[117] Agnati L.F., Guidolin D., Guescini M., Genedani S., Fuxe K. (2010). Understanding Wiring and Volume Transmission. Brain Res. Rev..

[118] Kaushalya S.K., Nag S., Ghosh H., Arumugam S., Maiti S. (2008). A High-Resolution Large Area Serotonin Map of a Live Rat Brain Section. Neuroreport.

[119] Coggeshall R.E., Fawcett D.W. (1964). The Fine Structure of the Central Nervous System of the Leech, Hirudo Medicinalis. J. Neurophysiol..

[120] Trueta C., Sánchez-Armass S., Morales M.A., De-Miguel F.F. (2004). Calcium-Induced Calcium Release Contributes to Somatic Secretion of Serotonin in Leech Retzius Neurons. J. Neurobiol..

[121] Reuter H. (1996). Diversity and Function of Presynaptic Calcium Channels in the Brain. Curr. Opin. Neurobiol..

[122] De-Miguel F.F., Santamaría-Holek I., Noguez P., Bustos C., Hernández-Lemus E., Rubí J.M. (2012). Biophysics of Active Vesicle Transport, an Intermediate Step That Couples Excitation and Exocytosis of Serotonin in the Neuronal Soma. PLoS ONE.

[123] Wojda U., Salinska E., Kuznicki J. (2008). Calcium Ions in Neuronal Degeneration. IUBMB Life.

